# Toward bioinspired polymer adhesives: activation assisted via HOBt for grafting of dopamine onto poly(acrylic acid)

**DOI:** 10.1098/rsos.211637

**Published:** 2022-03-30

**Authors:** Erik M. Alberts, P. U. Ashvin Iresh Fernando, Travis L. Thornell, Hannah E. George, Ashlyn M. Koval, Manoj K. Shukla, Charles A. Weiss, Lee C. Moores

**Affiliations:** ^1^ SIMETRI, 7005 University Blvd., Winter Park, FL 32792, USA; ^2^ Bennett Aerospace, 1100 Crescent Green, #250, Cary, NC 27518, USA; ^3^ Oak Ridge Institute for Science and Education, 1299 Bethel Valley Rd, Oak Ridge, TN 37830, USA; ^4^ US Army Engineer Research and Development Center, Geotechnical and Structures Laboratory, 3909 Halls Ferry Road, Vicksburg, MS 39180, USA; ^5^ School of Polymer Science and Engineering, The University of Southern Mississippi, 118 College Dr, Hattiesburg, MS 39406, USA; ^6^ US Army Engineer Research and Development Center, Environmental Laboratory, 3909 Halls Ferry Road, Vicksburg, MS 39180, USA

**Keywords:** activation, adhesion, catechol, bioin spired, poly(acrylic acid)

## Abstract

The design of bioinspired polymers has long been an area of intense study, however, applications to the design of concrete admixtures for improved materials performance have been relatively unexplored. In this work, we functionalized poly(acrylic acid) (PAA), a simple analogue to polycarboxylate ether admixtures in concrete, with dopamine to form a catechol-bearing polymer (PAA-g-DA). Synthetic routes using hydroxybenzotriazole (HOBt) as an activating agent were examined for their ability in grafting dopamine to the PAA backbone. Previous literature using the traditional coupling reagent 1-ethyl-3-(3-dimethylaminopropyl)-carbodiimide (EDC) to graft dopamine to PAA were found to be inconsistent and the sensitivity of EDC coupling reactions necessitated a search for an alternative. Additionally, HOBt allowed for greater control over per cent functionalization of the backbone, is a simple, robust reaction, and showed potential for scalability. This finding also represents a novel synthetic pathway for amide bond formation between dopamine and PAA. Finally, we performed preliminary adhesion studies of our polymer on rose granite specimens and demonstrated a 56% improvement in the mean adhesion strength over unfunctionalized PAA. These results demonstrate an early study on the potential of PAA-g-DA to be used for improving the bonds within concrete.

## 1. Introduction

Interest in the design of catechol-bearing polymers has rapidly exploded over the last decade across a diverse array of industrial and biomedical applications such as adhesives, energy storage and drug delivery platforms [1–3]. This research has been in part driven by the characterization of the foot proteins of mussels, such as *Mytilus edulis*, which contain an unusually high concentration of post-translationally modified tyrosine residues in the form of dihydroxyphenylalanine (L-DOPA) [4,5]. The catechol moiety of L-DOPA provides mussels with the ability to adhere to surfaces in wet environments, an elusive property for many synthetic materials [4,6–8]. Along with its wet-setting properties, mussel protein adhesion is not hindered by the surface energy of the substrate, as bonding has been demonstrated even to surfaces such as Teflon [4]. This versatility arises from its unique ability to bind to organic and inorganic substrates in multiples ways, including ionic coordination, π–π stacking, covalent and hydrogen bonding [9–11].

While mussel-inspired hydrogels, adhesives and coatings have gained extensive interest in biomedicine and nanotechnology [12–14], the use of such polymers in construction applications, such as concrete, has been relatively unexplored. When considering other marine adhesive-producing organisms, analogous comparisons to concrete have emerged. The eastern oyster*, Crassostrea virginica*, produces an organic–inorganic hybrid adhesive to adhere to one another forming vast reef structures [15,16] and incorporates material from the surrounding environment that enhances the material properties [17] in a similar fashion that aggregate can enforce cement paste. However, the understanding of oyster adhesion is incomplete, and only indirect evidence of DOPA chemistry has so far been found [18]. The sandcastle worm, *Phragmatopoma californica*, is known to secrete an L-DOPA containing silk-like adhesive to build habitats from sand grains [19–21].

These marine cementitious analogues suggest that such bioinspired polymers could be used in concrete for improved material properties. Polymers have been widely used as chemical admixtures in concrete, enabling enhanced properties such as setting, workability, durability and chemical resistance [22,23]. In particular, polycarboxylate ethers (PCEs) are employed as superplasticizers and set retardants in concrete and have been more recently investigated for use in low-carbon ‘green’ cements [24–26]. PCEs are primarily composed of carboxylic acid blocks that are frequently combined with polyethylene oxide side chains. In ordinary Portland cement, particles in solution tend to flocculate due to electrostatics; the PCEs acting as surfactants in concrete mixes can reduce the amount of water by chain adsorption onto charged cement particle surfaces to better disperse them. The addition of polymer chains deflocculates the hydrating cement particles by changing the overall charge (zeta potential) of the solution by electrostatics and by steric hindrance limiting the van der Waals forces between particles [27].

In addition to the applications mentioned above for polymeric admixtures, there is potential for adhesive polymers to be used in strengthening the interfacial transition zone (ITZ) between aggregate and paste [28,29]. In this region, differences in elastic modulus and shrinkage lead to crack formation, making the ITZ a significant weak point in the composite [30]. Catechol moieties could be useful in this region due to their ability to bind to inorganic surfaces such as those found of common aggregate substrates as well as with calcium-silicate-hydrate (CSH), the primary component of cement paste. In this work, we examine poly(acrylic acid) (PAA) as a simple PCE analogue in the synthesis of a catechol-bearing polymer with adhesive functionality via grafting with dopamine.

While PAA-dopamine (PAA-g-DA) conjugates have been described in prior literature, the methodology behind the synthesis has been inconsistent, and no reports on repeatability or scalability are mentioned [31–33]. Conjugation of primary amines with carboxyl groups to form amide linkages has traditionally been performed using carbodiimides, such as the zero-length cross-linker 1-ethyl-3-(3-dimethylaminopropyl)-carbodiimide (EDC) [34–36]. Water solubility and activation at physiological pH have earned EDC widespread use in conjugation reactions, peptide synthesis and peptide immobilization. In the coupling process, EDC forms an active ester intermediate, O-acylisourea, which directly interacts with an amine to form the amide bond and releases water-soluble urea by-product which can be difficult to separate out from water-soluble products on a larger scale. Despite this efficiency, EDC is susceptible to hydrolysis and has the tendency for formation of the non-reactive N*-*acylurea during coupling reactions [37,38]. An additional complication for PAA is the formation of anhydride during the EDC coupling process, preventing the formation of the amide bond [39].

The addition of N-hydroxysuccinimide (NHS) or N-hydroxysulfosuccinimide (sulfo-NHS) stabilizes the intermediate by the formation of an NHS-ester and has been shown to enhance amide bond yields [40]. The reaction is widely accepted as a two-step process, with the formation of the O-acylisourea optimized at pH 4.5–7.5 and the formation of the NHS-ester at pH 7.5–8. Alternative activators for EDC that focus on decreasing racemization of the acylurea have also been explored, including hydroxybenzotriazole (HOBt), which, alongside reducing racemization, increases the rate of the coupling reaction [41]. During the amide formation reactions, HOBt forms activated esters that react with amines at room temperature to give the desired amides. One drawback of anhydrous HOBt is its explosive potential in non-aqueous systems; however, most commercially available HOBt is provided wetted at no less than 20% water and does not exhibit explosive properties [42]. Additionally, gas-phase experiments showed triazole-ester reagents such as HOBt to be more reactive than NHS-esters and, by computational predictions, to have a lower transition state barrier [43].

In this work, the synthesis of PAA-g-DA in an aqueous solvent system was optimized by exploring an HOBt-based activation pathway without the use of EDC. Our goal was to synthesize a catechol-bearing polymer adhesive for use as a concrete admixture focusing on a simplistic reaction and have the potential for scale-up.

## 2. Material and methods

### 2.1. Reagents

High purity deionized water was obtained from a Millpore milli Q Advantage water system with a measured resistivity of ≥18.2 MΩ cm^−1^ (DI-water). Dopamine hydrochloride (DA) 98% (CAS# 62-31-7), N-(3-dimethylaminopropyl)-N′-ethylcarbodiimide hydrochloride (EDC) 98% (CAS# 25952-53-8), NHS 98% (CAS# 6066-82-6) and 1-HOBt 97% (CAS# 123333-53-9) were obtained from Sigma Aldrich (St. Louis, MO) and used as received. Triethylamine 99% (CAS# 121-44-8) was obtained from ACROS organics (Fairlawn, NJ). Poly(acrylic acid) 50 000 MW (PAA), 25% aqueous solution was obtained from Polysciences, Inc. (Warrington, PA). Deuterated water (D_2_O, 99.9%d) was obtained from Sigma Aldrich (St. Louis, MO).

### 2.2. Synthesis of dopamine-grafted poly(acrylic acid) via HOBt-mediated activation

**Scheme 1.**
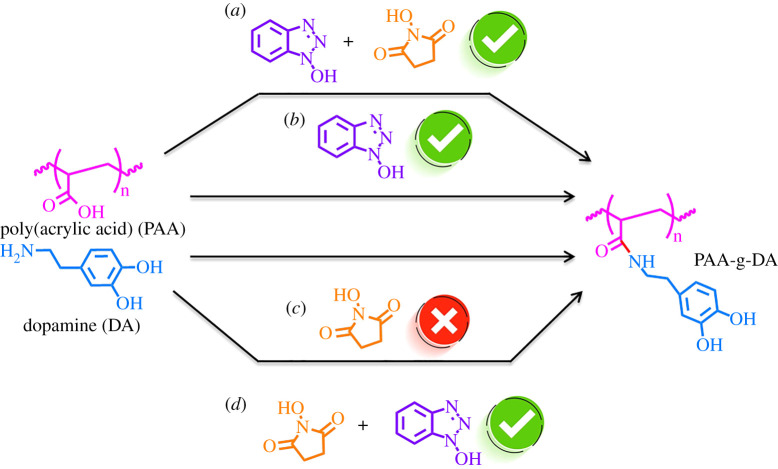
Synthetic scheme for various reactions routes that were performed in this work (*a*) HOBt added first followed by NHS, (*b*) HOBt alone, (*c*) NHS alone, and d) NHS initially added followed by HOBt. Green checkmarks indicate pathways with successful grafting of dopamine to PAA.

PAA (1.1 ml, 4.16 mmol) was diluted in DI-water (15 ml) and stirred while a stream of nitrogen bubbled through the solution for 10 min using a long needle (18 G) to remove dissolved oxygen and was kept constant throughout the reaction process. Grafting of dopamine to PAA was performed along the routes shown in scheme 1. First, either NHS (0.240 g, 4.16 mmol) or HOBt (0.675 g, 4.16 mmol) was added and stirred for 10 min. To aid the solubility of HOBt, it was first dissolved in a minimal amount of anhydrous N,N-dimethylformamide (DMF). For reactions featuring only HOBt or NHS, once the addition was complete, the solution was stirred for 15 min. After completion of the addition, white precipitate formed due to the insolubility of HOBt, and additional DMF (5 ml) was added. The additional DMF re-dissolved the HOBt, and the solution turned a pale yellow. For two of the synthetic routes, NHS and HOBt were used together, alternating what was added first. Finally, DA (0.789 g, 4.16 mmol) was added in small increments ensuring complete dissolution with each addition. The reaction was stirred for 10 min. The pH was adjusted with 100 µl of 1 M NaOH and a catalytic amount of trimethylamine (150 µl, 0.1 mol%) and stirred for 48 h at room temperature. Dialysis was performed for 3 days in DI water at room temperature, followed by lyophilization to obtain a solid white product. The above reaction has a stoichiometric ratio of 1 : 1 : 1 : 1 (PAA : NHS : HOBt : DA) for all reactants except catalytic amounts of NaOH and trimethylamine. Similarly, reactions were performed for 1 : 1 : 2 : 2 and 1 : 1 : 0.5 : 0.5. Additionally, reactions were conducted with and without the catalytic amounts of the base.

### 2.3. Instrumentation for characterization

A ThermoFisher Scientific (Waltham, MA, USA) Nicolet iS 10 FTIR spectrometer was used for FTIR characterizations. 1D ^1^H and ^13^C NMR were performed using a Bruker (Billerica, MA, USA) Avance 300 MHz NMR spectrometer, while 2D ^1^H-^1^H COSY and ^1^H-^13^C HMBC NMR were performed on a Bruker (Billerica, MA, USA) Avance 600 MHz NMR. All NMR spectra were collected in D_2_O. Thermogravimetric analysis was performed using a TA Instruments (New Castle, DE, USA) simultaneous thermal analyser (SDT 650) under nitrogen atmosphere. Data were collected from 30 to 800°C at a heating rate of 10°C min^−1^ after water was removed through equilibration at 110°C for 4 min. Scanning electron microscopy (SEM) images were obtained in backscatter mode using a Phenom ProX SEM (Phenom-World B.V., Eindhoven, Netherlands).

### 2.4. Adhesive testing

To evaluate the potential of PAA-g-DA as a bonding agent in concrete, tensile adhesion tests using a modified ASTM D2095 method were performed. Rose granite, selected as a model substrate for aggregate commonly found in concrete composites due to its relatively lower porosity than other aggregate materials (e.g. limestone), was cut into cylindrical specimens for butt-joint testing. Such testing allows for examination of uniaxial tension, which is relevant for strength testing of concrete materials, [44–46] especially along the ITZ where such forces cause crack propagation to occur. The adhesive was prepared in water at 0.3 g mL^−1^ and cured for 48 h at room temperature onto cylindrical rose granite adherends affixed to steel plantons with epoxy (Sikadur-35 Hi-Mod LV) (electronic supplementary material, figure S1). Adherends were polished using a Buehler Ecomet 3 variable speed grinder-polisher with P-120 grit silicon carbide paper. For each adherend, 50 µl of PAA-g-DA or PAA solution (0.3 g ml^−1^) was applied to the interface and a 250 g weight was used to apply pressure to the interface during curing. Adhesion tests were performed using an Instron (Norwood, MA, USA) universal testing system at 0.02 in min^−1^ with a preload force of 30 lbf. The reasoning behind the specific preload force was to rectify any residual kinks within the bicycle chain without masking the adhesives stress response. The bicycle chains allow for some rotational freedom but need some applied force to have the pins straighten out as displacement as tension is applied. The steel plantons were attached to the load frame with bicycle chains to allow for rotational freedom during loading.

## 3. Results and discussion

### 3.1. PAA-dopamine synthesis and comparison with literature

While PAA-dopamine has been synthesized via EDC-mediated coupling previously, [31–33,47] methodology has been inconsistent, as presented in table 1. While there are several reports on PAA and polydopamine, the selected articles were specific to PAA-dopamine synthesis via grafting. While this point is not discussed in prior work with PAA-dopamine, it was found that a nitrogen-purged solution and atmosphere through all steps were essential for the success of the coupling reaction. Dissolved oxygen may exhaust the coupling agent, and reaction yield drops significantly, often resulting in no coupling altogether.

**Table 1.** Reaction conditions for previously reported grafting of dopamine onto PAA compared with conditions used for present work.

article	PAA MW/mmol added	coupling reagents used/mmol added	dopamine/mmol added	reaction conditions	dialysis conditions	% grafting
present work	50 kDa – 4.16 mmol	^a^HOBt – 4.16 mmol (activating agent)	4.16 mmol	DI water + catalytic amount of NaOH/TEA N_2_ atmosphere	DI water, 72 h	8–18%
Min *et al.* [31]	50 kDa – mmol not specified	EDC – mmol not specified	not specified	pH 6.5, PBS buffer, atmospheric conditions not specified	Not specified	27.5%
Lee *et al.* [32]	100 kDa – 6.90 mmol	EDC – 1.39 mmol	0.69 mmol	pH 5.5, DI water, atmospheric conditions not specified	pH 5.0, 10 mM NaCl	7%
Wu *et al.* [33]	5 kDa – 3 mmol	EDC – 1.00 mmol Sulfo-NHS – 1.00 mmol	5.00 mmol	pH 6.0, 0.1 M PBS buffer, under N_2_	Milli-Q water	9%
Duan *et al.* [47]	450 kDa (Mn) 100 kDa (Mn) – mmol not specified	DCC – 10.00 mmol NHS – 10.00 mmol	not specified	PAA-NHS in DMF and PAA-Dopamine in pH 8.5 PBS buffer	No dialysis was conducted	450 kDa – 7% 100 kDa – Not specified

^a^Present work conditions are based on the 1 : 1 : 1 PAA : HOBt : DA reaction conditions.

The four articles cited in table 1 used a range of molecular weights of PAA, from 5 to 100 k. For this work, a 50 k MW PAA was used. Except for Duan *et al*. [47] EDC was used as the coupling reagent for grafting dopamine for all work, and only Wu *et al*. [33] used NHS as an activator. The schemes by Min *et al.* [31] Lee *et al.* [32] and Wu *et al.* [33] performed the reaction as a one-pot synthesis, whereas Duan isolated the PAA-NHS ester first from a *N,N’*-dicyclohexylcarbodiimide (DCC)-PAA reaction and then reacted the isolated product with dopamine. Reaction conditions were maintained in a pH range of 5.5–6.0 for EDC; however, according to the information provided in table 1, Lee *et al.* [32] did not use a PBS buffer. Dialysis conditions also varied; Lee *et al.* [32] used a pH 5.0, 10 mM NaCl dialysis, Wu *et al.* [33] used unadjusted Milli-Q water, and Min *et al.* [31] did not specify the conditions. The present study found no significant differences in grafting per cent as a result of using PBS buffer versus DI water during reaction or in dialysis conditions. However, dialysis was required for at least 3 days to entirely remove unreacted reagents and by-products, followed by lyophilization for 3 days to remove all residual water from the final fibrous product. Finally, % grafting, as determined by NMR integration of polymer backbone protons and aromatic protons was under 10% except for Min *et al*. who reported a high 27.5% functionalization. It is unclear why grafting yield was so much higher for the study by Min *et al.* [31] and the amount of dopamine used in the reaction was not provided. Inconsistency in amide bond yields with EDC and sensitivity to pH is problematic with replication and scalability of these reactions. Additionally, unwanted formation of anhydride with PAA and EDC adds additional complications to grafting yields.

Given these issues, an alternative to EDC was sought. In scheme 1, four alternative routes toward synthesis of PAA-g-DA are depicted. Additionally, we attempted the HOBt + NHS route both with and without a catalytic amount of base and triethylamine. For all coupling routes, reagents were added one at a time and allowed to stir in solution before additional reagents were added. HOBt required 1 : 1 ratio of DMF : water for complete dissolution. After the addition of dopamine, the reaction was allowed to stir for at least 48 h providing adequate time for coupling of the dopamine to the polymer. An additional 24 h was allowed for scaled-up reactions.

### 3.2. Validation and characterization of PAA-g-DA

**Figure 1.**
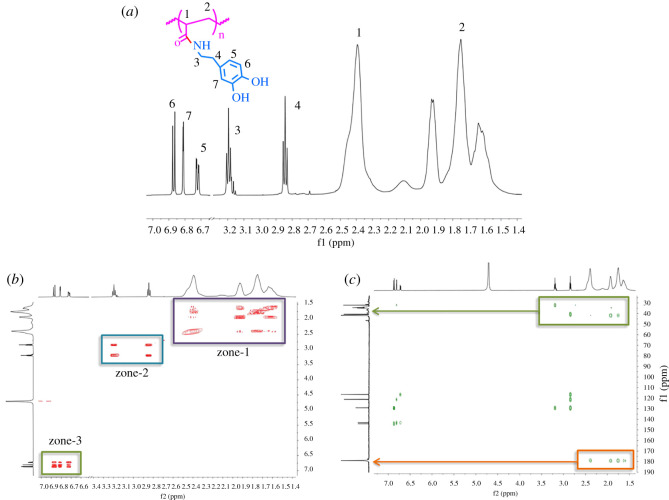
(*a*) ^1^H NMR (solvent peak omitted for clarity), (*b*) ^1^H-^1^H COSY spectra and (*c*) ^1^H-^13^C HMBC spectra of final functionalized polymer product. Spectra collected in 99.5% deuterium oxide.

After lyophilization, grafting of catechol was confirmed by the presence of aromatic protons in ^1^H NMR spectra (figure 1). The PAA backbone is characterized by a broad peak centred at 2.2 ppm arising from the de-shielded proton of the carbon adjacent to the carboxyl group, and a broad split peak around 1.5 ppm for the methylene protons. The methylene bridge of the dopamine functional group is seen as a pair of triplets near 2.8 and 3.15 ppm, shifted downfield from those in the dopamine starting material. The aromatic protons of the dopamine appear between 6.6 and 6.9 ppm as a series of doublets arising from ortho and meta coupling of the catechol [48]. Percentages of functionalization were calculated by the integration of the proton peak near the carbonyl group (labelled 1 in figure 1) of the polymer backbone to the aromatic proton isolated from the other aromatic peaks (labelled 5–7 in figure 1). From this integration ratio, the percentage of acrylic acid grafted with dopamine was derived. Additionally, it was evident from ^13^C NMR that the amide linkage has formed due to a peak around 177 ppm, which is typical for an amide carbonyl carbon (electronic supplementary material, figure S2). In addition, two-dimensional NMR further characterized the amide bond formation. Bond coupling was observed in HMBC spectra (figure 1*c*) through the carbonyl carbon of the amide bond with the polymer protons, indicating amide formation. Two-dimensional COSY NMR (figure 1*b*) showed that there is J^3^ coupling between the polymer protons (zone-1), followed by methylene proton coupling (zone-2), and finally aromatic protons coupling (zone-3).

**Figure 2.**
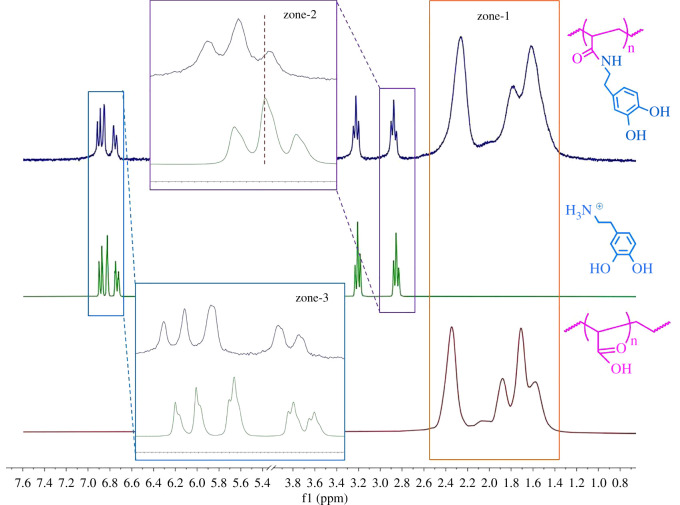
Stacked ^1^H NMR spectra of PAA, DA and PAA-g-DA.

As shown in figure 2, when the desired PAA-g-DA was formed, the methylene protons shifted more downfield (Δδ = 0.0145) when compared with the DA by itself. This again indicates that the amide bond was successfully formed, as the downfield shifts are due to the presence of carbonyl group from the polymer backbone. Similarly, the downfield shift was seen in the aromatic protons due to the electronegative effects from the amide bond formation (Δ*δ* = 0.0291). The aromatic protons after grafting were broadened due to faster transverse relaxations times (*T_2_*) when a small molecule such as dopamine is bound to the large macromolecule such as PAA.

**Figure 3.**
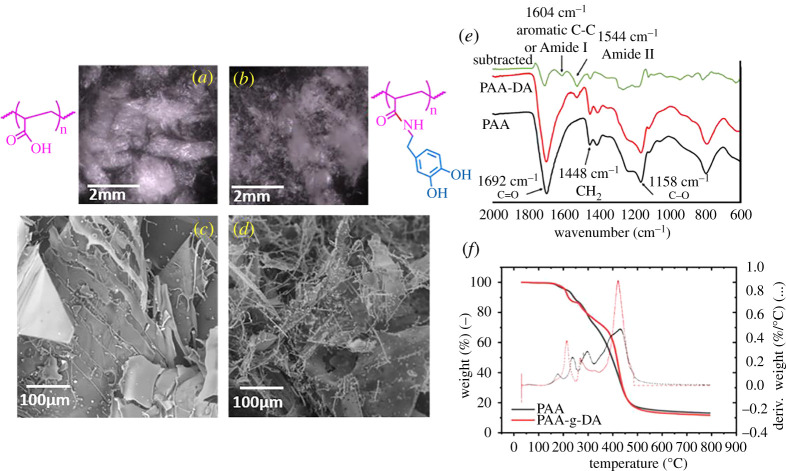
(*a*) and (*b*) Optical and (*c*) and (*d*) SEM micrographs of PAA and PAA-g-DA (8% functionalized) showing the change in morphology of the freeze-dried product. (*e*) FTIR spectra of PAA, PAA-dopamine, and the subtracted spectra. (*f*) TGA (solid) and DTG (dashed) of PAA and PAA-g-DA samples in a nitrogen atmosphere.

Optical and SEM imaging of PAA and PAA-g-DA (figure 3*a–d*) highlights the morphological changes of grafting dopamine to the polymer. The PAA stock was diluted prior to freeze-drying to match the concentration found in the reaction solution and appears to comprise thin, sheet-like structures with thin strands along the edges, probably an artefact arising from drying as water is pulled from the material. By contrast, the grafted polymer retained sheet-like morphologies but also contained many thin, fibrous structures. Additionally, the successful coupling can be confirmed by the presence of the amide bond in the FTIR spectrum (figure 3*e*) [49]. A small peak at 2934 cm^−1^ (figure 3*e* and electronic supplementary material, figure S3) corresponds to CH_2_ stretching vibration from the polymer backbone; as DA is grafted to the backbone the molecular weight increases, which results in a change of vibration inversely proportional to the change in mass. In the final product, this peak has shifted to 2930 cm^−1^, as the addition of dopamine increases molecular weight. The most prominent peak in the spectra is the carbonyl stretching frequency at 1694 cm^−1^ which shifts to 1697 cm^−1^ in the dopamine grafted product. Subtraction of the PAA spectra from the PAA-g-DA spectra resolves a hidden peak near 1604 cm^−1^ that may correspond to a portion of the amide I band or to aromatic C-C bonds. For amide I, this band contains characteristics of both the C=O and C-N stretches, leading to the overlap with the C=O of the neat PAA. The peak at 1544 cm^−1^, only present in PAA-g-DA, correlates to the amide II, confirming grafting of dopamine to PAA. Amide II contains characteristics of N-H bending and C-N stretching. Given there was only 8% grafting in the product analysed, it is expected for this peak to be small.

The TGA and differential (DTG) curves of 50 K MW PAA and the dopamine functionalized 50 K MW PAA are shown in figure 3*f*. The hydrophilic nature of PAA can cause large amounts of water (approx. 5–10 wt%) to be present that can obscure results, and so care was taken to evaporate water by equilibrating the material for 4 min at 100°C. The first region of 140–310°C had an observed weight loss of 25% for all samples; previous reports have linked degradation in this region to carboxylic acid side chains interactions and decomposition, cyclization to form anhydrides, decomposition and release of CH_4_ and CO_2_ [50,51]. The main chain degradation and scission of PAA is the second region of temperatures above 310°C with a mass loss of 57.41% for the 50 K MW PAA control. The dopamine-containing PAA (PAA-g-DA) had similar thermal decomposition behaviour. In the first region of interest at 140–310°C, PAA-g-DA exhibited a 21.12% weight loss. It appears that dopamine slightly enhances the stability in the second region of 310–600°C that has been previously attributed to the main chain degradation. In the range of 300–400°C, the PAA-g-DA showed a delay in degradation compared with non-functionalized PAA. Catechol groups improving the thermal stability of polymers has been observed in prior literature and has been attributed to the hydrogen bonding and restriction of chain motion [52,53]. Additionally, dopamine can scavenge radicals generated by C-C bond pyrolysis, blocking depolymerization of the PAA backbone by chain scission [52,54]. At a temperature of 600°C, the 50 K MW PAA has reached an equilibrium weight of 19.0% remaining. For the dopamine functionalized samples, the weight remaining at 600°C was 17.9%. This may indicate the breakdown of the styrenic rings and other oxidized structures.

### 3.3. Effect of HOBt and NHS on coupling

**Figure 4.**
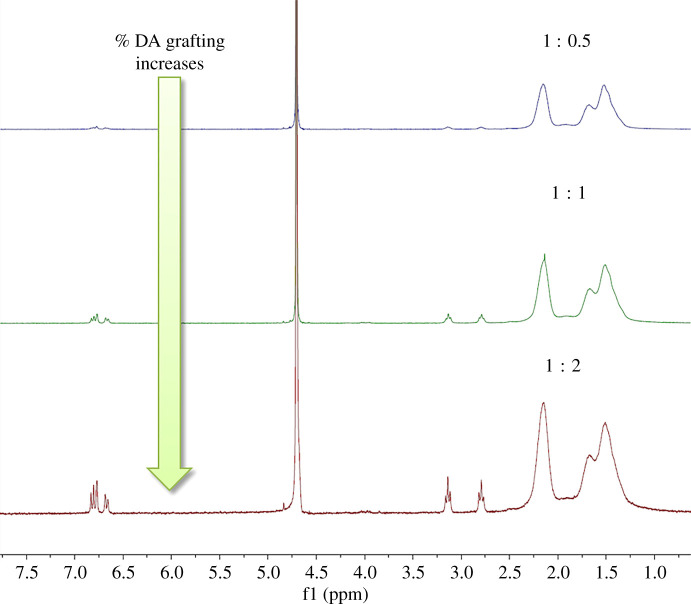
^1^H NMR stacked spectra of varying equivalents of DA for the grafting reaction.

For all experiments, HOBt-mediated grafting resulted in a soluble product that showed a coupling dependence on the ratio of the dopamine/activating reagent to polymer used. As this ratio increased, so did the percentage of grafting (figure 4). In table 2, grafting percentage for the 1 : 1 : 1 (PAA : HOBt : dopamine) reactions for each of the four pathways tested are shown. When in the presence of NHS, the reaction seemed to perform less efficiently (8% grafting) when compared with when HOBt is the sole activating agent (11% grafting). This is potentially due to the two reagents acting as competitive activation reactions resulting in lower dopamine grafting yields. For the reaction with NHS without HOBt, the polymer product showed signs of dopamine oxidation, evidenced by dark material formation after dialysis. Oxidation of catechol leads to a higher degree of cross-linking within the polymer resulting in lower adhesive strength, and so was discarded [55]. Addition of a catalytic amount of base to the reaction improved grafting yield for path b (HOBt) to 18% and path d (NHS + HOBt) to 12%, while path a (HOBt + NHS) was unchanged. It was also found that repeated experiments were consistent in grafting yield within a range of a few per cent and the order of adding NHS and HOBt was largely irrelevant to the final grafting yield. To further test the robustness of the reaction, the synthesis of the HOBt + NHS route at 2×, 6× and 12× scale-up based on the reagent amounts described in the methods was conducted (§2.2) (table 3). The HOBt pathway continued to show improved grafting over the NHS + HOBt pathway; however, the grafting percentage did decline from 18% to 11–13% at the 6× and 12× scale, respectively. Overall, this indicates that the use of HOBt as the sole activating agent increases the reaction performance as there is no other competitive activating agent which could hinder the reaction efficiency through a competitive process.

**Table 2.** Grafting percentage for the coupling pathways used in these experiments, both with and without a catalytic amount of base. Ratios of all reagents were 1 : 1 for all reactions.

path used	grafting % with base	grafting % without base
path a HOBt + NHS	8	8
path b HOBt	18	11
path c NHS	oxidized product	oxidized product
path d NHS + HOBt	12	8

**Table 3.** Scale-up of the HOBt-mediated coupling for 1 : 1 : 1 ratio reactions.

scale	grafting % NHS HOBt	grafting % HOBt
1×	10	18
2×	12	18
6×	10	11
12×	9	13

### 3.4. Proposed mechanism for HOBt-mediated synthesis

**Figure 5.**
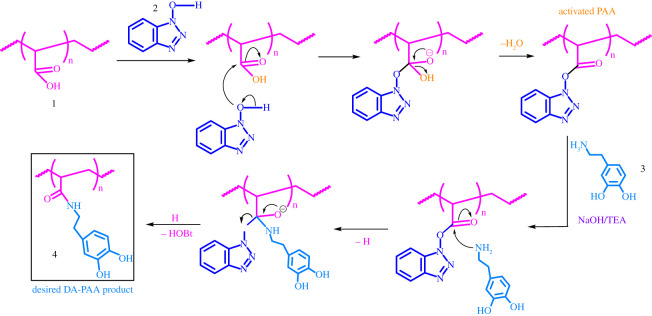
Proposed mechanistic route for HOBt-mediated synthesis.

HOBt has traditionally been used in combination with a carbodiimide coupling agent, like EDC, to enhance peptide coupling reactions (vide supra) [56]. However, our experimental results show the reaction can take place without the carbodiimide. The utilization of HOBt as an activating agent appears to be an alternative novel method for functionalizing PAA with amide moieties. Shown in figure 5, we propose that the activation is initiated by condensation between the carboxylic acids on PAA with the HOBt leading to the formation of activated transesterified HOBt ester. The formation of free amine occurs by the addition of catalytic amounts of 1 M NaOH and triethylamine (TEA) [57]. The free dopamine reacts with the activated carboxylic acid while eliminating HOBt, resulting in the final desired dopamine grafted polyacrylic acid.

Reaction mechanism calculations were performed for the addition of dopamine to form the tetrahedral intermediate using M06–2X/6-31G(d) including implicit solvent (SMD = water). The PAA was modelled as a monomer unit truncated with a CH_2_CH_3_ group to save computational time. This computational modelling suggested that formation of the activated PAA-HOBt complex was unfavourable (electronic supplementary material, figure S5) despite experimental evidence; however, these results were simulated without accounting for the influence of DMF or the catalytic amount of base. Calculations on addition of dopamine to the already activated PAA showed the reaction barrier for PAA-HOBt (3.9 kcal mol^−1^) to be slightly less than the alternative PAA-NHS complex (4.3 kcal mol^−1^) (electronic supplementary material, figure S6), which indicates that the HOBt-activated pathway is more favourable than NHS-activated pathway. However, the final product for the HOBt pathway was only 0.1 kcal mol^−1^ lower than the predicted tetrahedral transition state complex which indicates this reaction is possibly reversible. Complementary work by Bu *et al.* [43] performed similar calculations of a sulfo-benzoyl-HOAt complex reacting with methylamine (NH_2_-CH_3_); reactants involved are different from those presented in this work's calculations and were done at a different level of theory [B2LYP/6-311G + +(d,p)]. The results discussed in Bu *et al.* [43] should and do differ from present work, but qualitatively similar trends were observed. The HOAt complex shown in Bu *et al.* [43] has a slightly lower activation barrier than the NHS complex, which aligns with data given in this study; however, the product provided in the SI of Bu *et al.* [43] is not a tetrahedral intermediate but optimized to where the HOAt has already eliminated from the intermediate. The conclusion is that rather than existing as an electrostatic, tetrahedral complex, the intermediate predicted in SI figure breaks relatively weak van der Waals interactions to yield the final product. Therefore, it is possible that allowing the tetrahedral intermediate to optimize and allow the HOBt to leave will then indeed make the overall reaction energetically favourable as shown in figure 5.

### 3.5. Adhesion testing of PAA-g-DA

**Figure 6.**
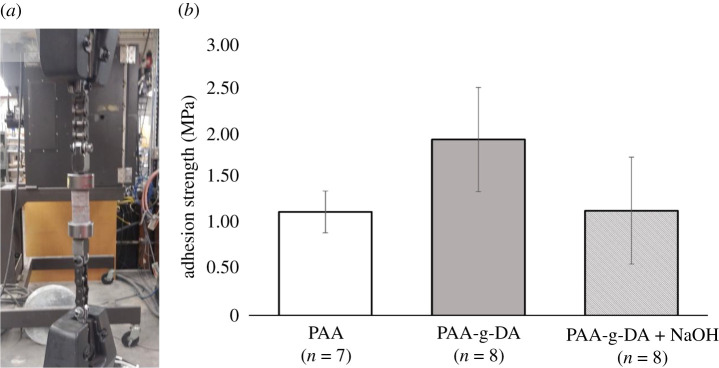
(*a*) Butt-joint adhesion testing with rose granite using an Instron material testing system. (*b*) Tensile adhesion strength for PAA, PAA-g-DA and PAA-g-DA with NaOH. Error bars as displayed are standard deviations from the mean tensile strength.

All samples were polished to reduce potential mechanical interference from rough surfaces. Figure 6*a* shows the modified ASTM D2095 set-up with rose granite substrates adhered to one another in tension. PAA and PAA-g-DA were fashioned as water-based adhesive to mimic the wet conditions found within concrete. The average tensile adhesive strength of 8% dopamine-grafted PAA (based on NMR analysis) was 1.94 MPa, significantly higher than PAA alone 1.15 MPa (*p*-value 0.0045). Adhesion strength for the PAA-g-DA showed more variability than PAA, with a maximum and minimum strength of 2.6 and 1.3 MPa, respectively, for the 10 samples examined (two PAA-g-DA adherends failed prior to loading). Most samples demonstrated cohesive failure, with PAA-g-DA visible on both adherends after the test (electronic supplementary material, figure S7A-B). Additionally, there were some instances of substrate failure, where the granite was chipped near the surface (electronic supplementary material, figure S7C). While the rose granite samples were polished, the porosity and inhomogeneity of the surface may have led to the variation seen in these results.

Adhesive strength was also tested for PAA-g-DA at a pH of 13–14 (comparable to that of concrete) with the addition of 10 M NaOH immediately following application of the polymer to the granite substrate. It was found that adhesion strength was considerably weakened in this condition, and while the maximum adhesion was higher than that of PAA alone (2.3 versus 1.3 MPa) the mean value was not statistically different to that of PAA alone (1.16 MPa, *p-*value 0.9569). The effect of pH on catechol adhesion in mussel foot proteins has been noted before, [58,59] with observations that oxidation of OH groups at high pH generally lead to weaker adhesive strength on mica and titanium. However, Yu *et al*. also noted that binding strength of the DOPA-containing mussel foot protein-3 increased on TiO_2_ with increasing pH (up to 7.5), due to a shift in binding mode from hydrogen to coordination bonding [60]. This increase in pH led to the opposing effects of decreasing DOPA-mediated adhesion and increasing bidentate DOPA-Ti coordination as a result of catechol oxidation [60]. We believe our study is the first on catechol adhesion at high alkaline conditions.

Finally, to examine the effect of moisture, adhesion tests were run at room temperature in high humidity environment (*R*_h_% = 70–80%) at both the low and high pH conditions. All specimens for this method failed at low loading, however, due to incomplete curing (electronic supplementary material, figure S8). Hydration of the material would lead to adhesive bond disruption, weakening the material. While we theorize that when used within concrete the adhesive will cure as the concrete dries, it may still be necessary to further optimize this system.

## 4. Conclusion

We sought to find a simple, robust and scalable route to synthesize dopamine-grafted PAA adhesive for use in concrete admixture. The work here illustrates a viable route for HOBt-mediated amide coupling of dopamine to PAA. The developed methodology avoids the unwanted by-products and sensitivity of EDC and provides greater control over the per cent DA grafted to the polymer. While HOBt is typically used as an activator with EDC [56], observations that HOBt can provide amide bond formation without EDC were made. Furthermore, HOBt alone aids in an unhindered activation-mediated reaction that assists in forming products without the use of an additional coupling agent. The synthetic scheme was robust and consistent, even when scaling the reaction up to 12-fold. Characterization through NMR and FTIR confirmed the formation of PAA-g-DA, and adhesion testing on rose granite substrate demonstrated successful bonding of the polymer to aggregate material, with a 56% improvement in adhesive strength over neat PAA. While further optimization of the material may be necessary for higher pH conditions, these results demonstrate a promising application for this material. Future work will focus on further adhesion testing between cement paste and aggregate substrates to simulate the bonding at the ITZ.

## Data Availability

All relevant data for this research are presented in this paper and electronic supplementary material [61]. Raw data files pertaining to instrument data from FTIR, TGA, NMR and tensile test measurements, as well as standard optimized coordinate files for computational work, are available from the Dryad Digital Repository: https://doi.org/10.5061/dryad.3bk3j9kmn [62].
